# Gadd45a deficiency accelerates BCR-ABL driven chronic myelogenous leukemia

**DOI:** 10.18632/oncotarget.14580

**Published:** 2017-01-10

**Authors:** Kaushiki Mukherjee, Xiaojin Sha, Andrew Magimaidas, Silvia Maifrede, Tomasz Skorski, Ravi Bhatia, Barbara Hoffman, Dan A. Liebermann

**Affiliations:** ^1^ Fels Institute for Cancer Research and Molecular Biology, Philadelphia, PA, USA; ^2^ Department of Microbiology and Immunology, Temple University, Philadelphia, PA, USA; ^3^ Department of Systems Pharmacology and Translational Therapeutics, Perelman School of Medicine, University of Pennsylvania, Philadelphia, PA, USA; ^4^ Division of Hematology and Oncology, University of Alabama, Tuscaloosa, AL, USA; ^5^ Department of Medical Genetics and Molecular Biochemistry, Temple University, Philadelphia, PA, USA

**Keywords:** Gadd45a, chronic myelogenous leukemia, stress response protein, tumor suppressor

## Abstract

The Gadd45a stress sensor gene is a member in the Gadd45 family of genes that includes Gadd45b & Gadd45g. To investigate the effect of GADD45A in the development of CML, syngeneic wild type lethally irradiated mice were reconstituted with either wild type or *Gadd45a* null myeloid progenitors transduced with a retroviral vector expressing the 210-kD BCR-ABL fusion oncoprotein. Loss of *Gadd45a* was observed to accelerate BCR-ABL driven CML resulting in the development of a more aggressive disease, a significantly shortened median mice survival time, and increased BCR-ABL expressing leukemic stem/progenitor cells (GFP+Lin- cKit+Sca+). GADD45A deficient progenitors expressing BCR-ABL exhibited increased proliferation and decreased apoptosis relative to WT counterparts, which was associated with enhanced PI3K-AKT-mTOR-4E-BP1 signaling, upregulation of p30C/EBPa expression, and hyper-activation of p38 and Stat5. Furthermore, Gadd45a expression in samples obtained from CML patients was upregulated in more indolent chronic phase CML samples and down regulated in aggressive accelerated phase CML and blast crisis CML. These results provide novel evidence that Gadd45a functions as a suppressor of BCR/ABL driven leukemia and may provide a unique prognostic marker of CML progression.

## INTRODUCTION

The GADD45 family of proteins (GADD45A, GADD45B, and GADD45G) act as stress sensors in response to various physiological and environmental stressors, including oncogenic stress [1–5] Gadd45 function is mediated *via* interaction of its cognate protein with partner proteins including PCNA, cdk1/cyclinB1 complex, p21, MEKK4, MKK7, and p38, to modulate cell cycle regulation [6] [7], DNA replication/repair [8, 9] [10] and cell survival [11]. Notably, in mammary breast cancer models, *Gadd45a* behaves as a tumor suppressor in response to H-RAS, and as an oncogene in response to Myc [12]. Additionally, *Gadd45a* and *Gadd45b* have been implicated in modulating the response of hematopoietic cells to haematological stressors *via* distinct signaling pathways, including p38 activation and JNK inhibition [13, 14]. Although members of the Gadd45 family seem infrequently mutated in cancer their reduced expression due to promoter methylation is observed in several types of human cancers [2] [15, 16]. Recently, while this work was in progress, it was shown that *Gadd45a* is a repressed target of activated FLT3 [17], and that *Gadd45a* promoter methylation is predictive of poor prognosis in AML [18].

The Philadelphia chromosome (Ph) arises from a balanced translocation involving chromosomes 9 and 22 [19]. This translocation forms the fusion oncoprotein, a constitutionally active tyrosine kinase BCR-ABL, which has been causatively linked to the development of Chronic Myelogenous Leukemia (CML). CML is characterized by the progression from an indolent ‘chronic phase’ (CML-CP), a phase in which mature granulocytes hyperproliferate, to the aggressive and fatal ‘blast crisis’ (CML-BC) marked by the clonal expansion of differentiation-arrested immature blasts [20–22].

Imatinib, a small molecule ABL kinase inhibitor is highly effective in treating CML-CP patients [23]. However, a substantial number of patients relapse due to development of resistance to imatinib therapy that leads to CML-BC, which is invariably fatal within weeks to months [24]. Thus, identification of additional genetic aberrations that play a role in the progression of CML is of utmost importance from a therapeutic point of view.

In this study we used the bone marrow transplantation (BMT) mouse model of CML to ask if and how Gadd45a modulates CML progression. Furthermore, the expression of Gadd45a was determined using samples from CML patients at various progressive stages of the disease. Collectively our data provides first evidence that gadd45a functions as a suppressor of BCR/ABL driven leukemia and may provide a novel prognostic marker of CML progression.

## RESULTS

### *Gadd45a* deficiency accelerates the onset of BCR-ABL driven leukemia in recipient mice

**Figure 1:**
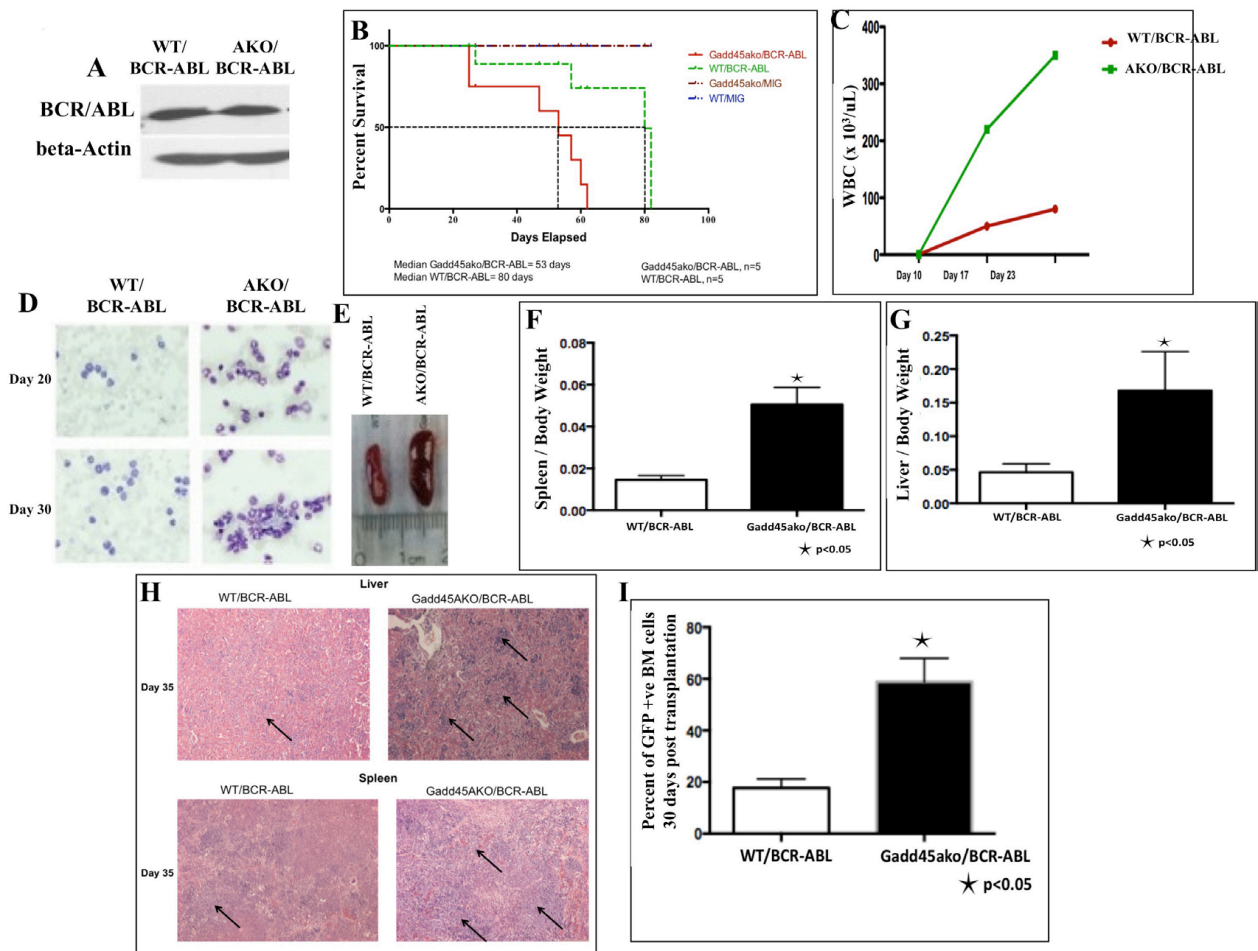
Loss of *Gadd45a* accelerates the onset of BCR-ABL driven leukemia in recipient mice **A**. There is no significant difference in expression of BCR-ABL in WT and *Gadd45a*−/− BM (AKO) cells used for BMT. **B**. Kaplan Meier survival curve of WT recipients transplanted with equivalent number of BCR-ABL transduced BM cells from the two genotypes (*n* = 5 per genotype, *P* < 0.05) **C**. Total number of WBCs in peripheral blood at indicated times after transplantation. (*n* = 3) **D**. May Grunwald Giemsa staining of peripheral blood 20 and 30 days after transplantation (original magnification, x600) **E**. Gross appearance of the spleens and **F**.-**G**. Ratio of spleen and liver weights to body weight 35 days post transplantation. Error bars represent SEM *p* < 0.05 (*n* = 3) **H**. H&E staining of liver and spleen sections revealed increased leukemic cell infiltration in mice transplanted with *Gadd45a*−/−/BCR-ABL-expressing BM (see arrows) **I**. Increased percentage of GFP+ve BM cells in mice transplanted with BCR/ABL-expressing *Gadd45a*−/− BM. Results are the average of 3 independent experiments.

To investigate the effect of loss of *Gadd45a* on BCR-ABL driven leukemia, BMT using WT and *Gadd45a* knockout (KO) bone marrow (BM) cells transduced with the BCR-ABL oncoprotein was performed. Infected BM used for BMT was found to express similar levels of BCR-ABL protein regardless of *Gadd45a* status (Figure 1A)

All mice that received transplants of BCR-ABL infected BM cells developed fatal haematological disease 11 weeks after BMT with evidence of enlarged liver and spleen resembling CML like disease. More importantly, mice transplanted with *Gadd45a*−/−/BCR-ABL myeloid progenitors exhibited reduced disease latency with a median of 53 days compared to WT/BCR-ABL recipients with a median of 80 days (Figure 1B).

White blood cell (WBC) counts in peripheral blood were significantly increased in *Gadd45a*−/−/BCR-ABL recipients compared to WT/BCR-ABL recipients (Figure 1C), and hematopathological analysis revealed this was associated with a dramatic increase in number of dysplastic granulocytes (Figure 1D). Additionally, *Gadd45a*−/−/BCR-ABL recipients exhibited massive splenomegaly and hepatomegaly compared to WT/BCR-ABL recipients due to increased extramedullary hematopoiesis (Figure 1E-1G). Histopathological analysis of H&E stained liver and spleen sections showed increased myeloid infiltration, indicative of more aggressive disease development in *Gadd45a* null background. (Figure 1H). Importantly, a dramatic increase in total number of BCR-ABL expressing cells in the BM of *Gadd45a*−/−/BCR-ABL mice (60% GFP+ve cells) was observed compared to WT/BCR-ABL recipients (20% GFP+ve cells) (Figure 1I), consistent with increased extramedullary hematopoiesis. Collectively, these observations are consistent with reduced disease latency and more aggressive leukemia in *Gadd45a*−/−/BCR-ABL mice, pointing to a potent and novel tumor suppressor role for *Gadd45a* in BCR/ABL driven leukemogenesis.

### *Gadd45a* expression is increased in BCR-ABL expressing BM

**Figure 2:**
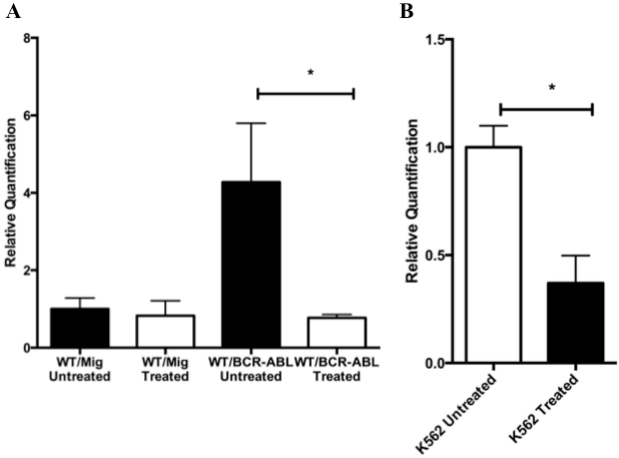
BCR-ABL upregulates *Gadd45a* expression **A**. Real time PCR analysis using taqman probe, of *Gadd45a* mRNA transcripts in WT BM cells transduced with either MIG or BCR-ABL expressing retroviral vector that has been treated or untreated with 5uM Imatinib. Expression is relative to WT/MIG treated sample. **B**. Real time PCR analysis using taqman probe, of *Gaddd45a* mRNA transcripts in K562 cells treated and untreated with Imatinib. Expression was determined by using 18s rRNA as an endogenous control in AB Step one plus real time PCR machine. Data from 3 experiments is shown. Error bars represent SD for each group (* *P* < 0.05).

Given the role Gadd45 proteins play as sensors of oncogenic stress and their regulated expression during development of hematopoietic cells, we assessed *Gadd45a* expression in wild type BM expressing the BCR-ABL oncoprotein. *Gadd45a* expression was found to be similar in the absence of BCR-ABL in both WT-MIG untreated and WT-MIG imatinib treated BM samples (Figure 2A). However, WT/BCR-ABL cells expressed significantly increased *Gadd45a* compared to WT-MIG cells. In the presence of Imatinib, which is a BCR-ABL tyrosine kinase inhibitor, *Gadd45a* expression was reduced to baseline levels, showing that BCR/ABL is responsible, either directly or indirectly, for increasing Gadd45a expression. Consistent with this conclusion, K562 cells that constitutively express BCR-ABL also express elevated *Gadd45a*, which is significantly reduced following treatment with Imatinib (Figure 2B).

### Loss of *Gadd45a* in myeloid progenitors expressing BCR-ABL enhances proliferation and reduces apoptosis

**Figure 3:**
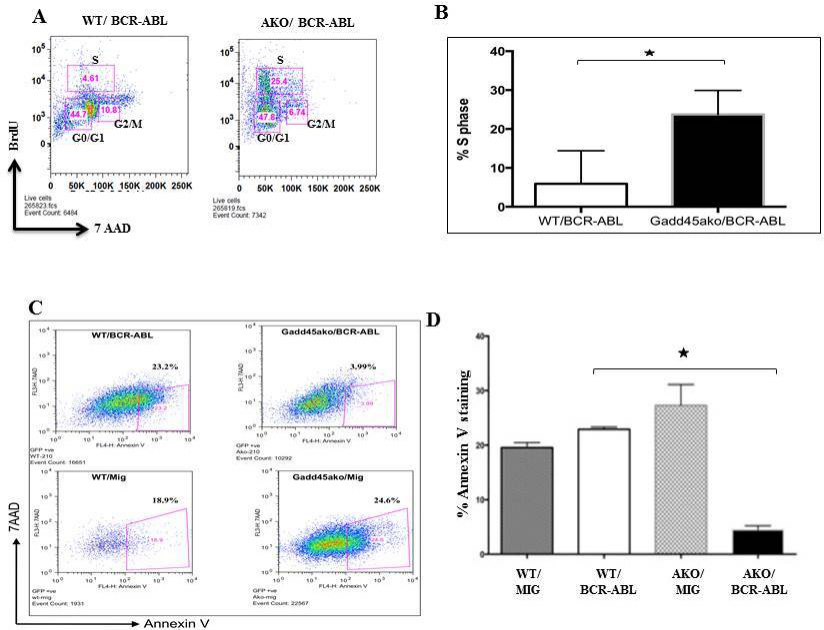
Loss of *Gadd45a* enhances proliferation and reduces apoptosis of myeloid progenitors expressing BCR-ABL oncoprotein **A**. Analysis of BrDU incorporation in BM cells from mice following BMT with *Gadd45a*−/−/BCR-ABL and WT/BCR/ABL BM, 35 days post transplantation. Cells undergoing proliferation showed an increase in % S-phase population. **B**. Quantification of percentage of S phase cells in WT/BCR-ABL and *Gadd45a*−/−/BCR-ABL BM from **A**.. Error bars represent SEM, *P* < 0.05 (*n* = 4 per genotype). **C**. Flow cytometric data of apoptotic events (annexin V incorporation) in WT/MIG, WT/BCR-ABL, *Gadd45a*−/− /MIG and *Gadd45a*−/−/BCR-ABL BM cells 4 days post retroviral infection. Early apoptotic cells are visualized in lower right quadrant. Data is representative of 3 independent experiments. **D**. Quantification of percentage of Annexin V positive cells from **C**.. Summary of FACS data showing average of 3 independent experiments ± SEM *P* < 0.05.

To understand how loss of Gadd45a accelerates disease initiation and progression we next asked whether loss of *Gadd45a* in the presence of BCR-ABL results in changes in the proliferation and apoptosis of BCR-ABL expressing cells. To this end, CML mice 35 days post BMT were intra-peritonially injected with BrdU and 24 hours later the BM was analyzed. FACS analysis showed an increase in cell numbers in S phase in *Gadd45a*−/−/BCR-ABL recipients compared to WT counterparts. (Figure 3A, 3B)

The effect of loss of Gadd45a on apoptosis in BCR/ABL expressing BM was assessed using annexin V analysis of WT and *Gadd45a*−/− BM cells, infected with either empty vector or BCR/ABL expressing vector. Early apoptotic events, measuring the percent 7AAD^-^AnnexinV^+^ cells, showed that 23.2% of the cells were in early stage of apoptosis for WT/BCR-ABL BM compared to only 3.99% of the cells in *Gadd45a*−/−/BCR-ABL BM (Figure 3C-3D). Intriguingly, loss of *Gadd45a* was also observed to prolong factor independent survival of BCR-ABL expressing Lin-ckit+Sca- progenitor cells (data not shown). The increased proliferation and decreased apoptosis observed in BCR/ABL expressing BM null for Gadd45a could play a role in the decreased latency of CML caused by loss of *Gadd45a*.

### Loss of *Gadd45a* leads to increased number of BCR-ABL expressing leukemic stem cells (LSCs) as well as an increase in self-renewal function of BM cells

**Figure 4:**
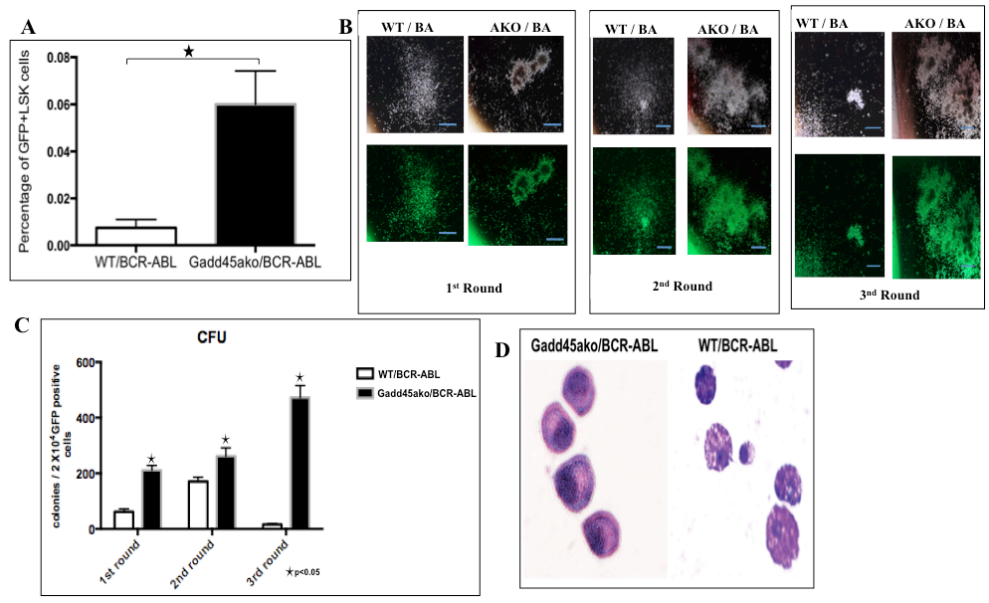
Loss of *Gadd45a* leads to an increase in the number of BCR-ABL expressing leukemic stem cells (LSCs) as well as an increase in BM self-renewal **A**. FACS analysis of GFP+Lin-Sca+cKit+ (LSK) cells in BM of *Gadd45a*−/−/BCR-ABL and WT/BCR-ABL mice respectively, 21 days post transplantation. Quantification of GFP+ cells that are LSK. Summary of FACS data showing average of 3 independent experiments Bars ± SEM, *P* < 0.05 **B**. Representative photomicrograph of colonies from WT/BCR-ABL and *Gadd45a*−/−/BCR-ABL BM cells from 3 rounds of serial replating. 10 X magnification. **C**. Average CFU per round of serial replating experiments. Bars ± SEM, *P* < 0.05 **D**. Photomicrograph of cytospins of *Gadd45a*−/−/BCR-ABL and WT/BCR-ABL cells from colonies (40X magnification). Data shown represents 3 independent experiments.

Since CML is a clonal hematopoietic stem cell disorder it was asked if the functional consequence of *Gadd45a* loss in CML is reflected by an increase in cells that are high up in the hierarchy of the hematopoietic system. To assess this, Lineage (Lin) negative spleen cells from CML mice were labeled with stem cell markers Sca and Kit, and it was observed that the percentage of GFP+Lin^-^cKit+Sca+ (LSK) cells in the *Gadd45a* deficient BCR-ABL expressing mice were significantly increased compared to WT/BCR-ABL mice (Figure 4A). These results are indicative of a higher proliferative capability of BCR-ABL expressing leukemic stem cells lacking *Gadd45a*.

To quantify the self-renewal ability of LSC, colony forming assays were carried out. *Gadd45a*−/−/BCR-ABL BM cells exhibited significantly increased self-renewal capability compared to WT/BCR-ABL BM cells (Figure 4C). In addition, *Gadd45a*−/−/BCR-ABL BM cells formed compact and undifferentiated colonies; in contrast to the more diffuse colonies formed by WT/BCR-ABL BM cells, indicative of less differentiated status of cells (Figure 4B). The morphology of the cells from the colonies was assessed using May Grunwald Giemsa staining of cytospin smears. WT/BCR-ABL cells were amorphous shaped with large cytoplasmic to nuclear ratio and protruding appendages, indicative of differentiation, whereas cells from *Gadd45a*−/−/BCR-ABL colonies were less differentiated, consistent with a stem/progenitor nature (Figure 4D).

Taken together, reduced disease latency in *Gadd45a*−/−/BCR-ABL transplanted mice is associated with an increase in the number of leukemic stem cells as well as an increase in the self-renewal capability and blocked differentiation of *Gadd45a* null BM expressing BCR/ABL.

### Loss of *Gadd45a* does not affect the number of normal hematopoietic stem cells as well as homing capability and repopulation ability of BM

**Figure 5:**
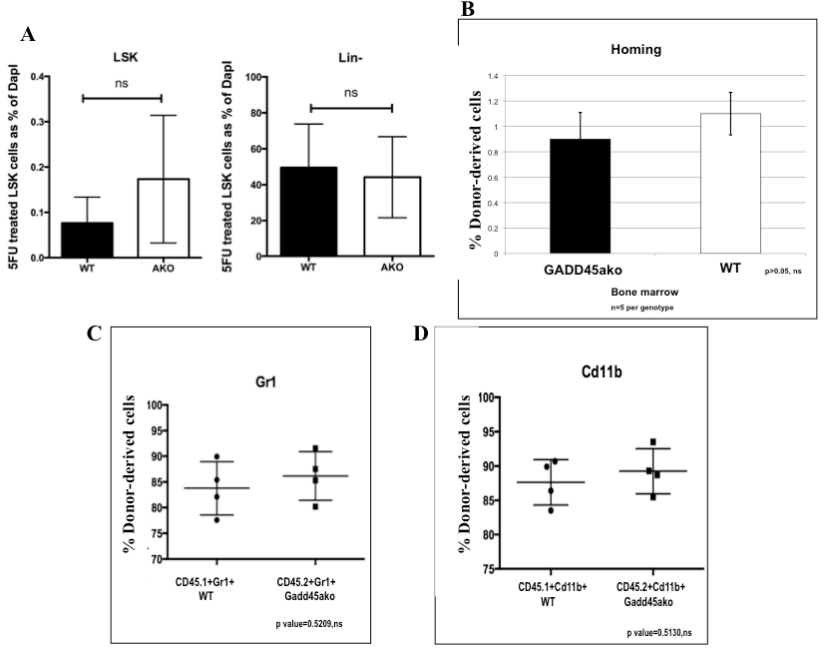
Loss of *Gadd45a* does not affect the number of Normal Hematopoietic stem cells as well as homing and engraftment of bone marrow cells **A**. Bar graph representation of flow cytometric data showing average LSK cells from 6 independent experiments. Differences between normal stem cells in WT and *Gadd45a*−/− mice were not significant. *P* > 0.05 **B**. FACS of donor derived CD45.2 (WT or *Gadd45a*−/−) cells in CD45.1 mice, 3 hrs post BMT. Bar graph shows average percent CD45.2 cells from 3 independent experiments (*n* = 5 per genotype). Bars ± SEM *P* > 0.05 **C**.&**D**. Summary of flow data from competitive repopulation experiments showing Gr1 and CD11b myeloid markers CD45.1+Gr1+, CD45.2+Gr1+ve, CD45.1+cd11b+ and CD45.2+cd11b+ve cells. *P* > 0.05 Results are representative of 3 independent experiments in each group (*n* = 4 per genotype).

It was next asked if the accelerated CML development caused by loss of *Gadd45a* is a reflection of its impact on either the hematopoietic stem cell (HSC) population or homing ability. No significant differences were detected in Lin- and LSK cells in BM from 5 FU treated *Gadd45a*−/− and WT mice (Figure 5A). Furthermore, 3 h after the transplantation of either WT of *Gadd45a*−/− BM cells into recipient mice (CD45.1), the percentages of donor-derived CD45.2 BM cells in recipient mice were similar (Figure 5B), indicating that *Gadd45a* deficiency does not significantly alter the homing ability of BM cells.

To further confirm that *Gadd45a* deficiency does not affect normal HSC function, a competitive reconstitution analysis was carried out. WT and *Gadd45a*−/− BM cells from mice expressing CD45.1 and CD45.2 cells, respectively, were mixed at 1:1 ratio (5 X105 per genotype) and transplanted into lethally irradiated CD45.1 recipient mice. 4 weeks post BMT, bone marrow cells were extracted from recipient mice and subjected to FACS analysis which revealed that the average percentage of donor derived CD45.1+Gr1+ and CD45.2+Gr1+ cells was 83% and 85%, respectively (Figure 5C) while the average percentage of CD45.1+cd11b+ and CD45.2+cd11b+ was 87% and 89%, respectively (Figure 5D). These results indicated no significant differences between CD45.1 and CD45.2 leukocyte derived myelopoiesis, indicating that *Gadd45a* deficiency does not provide a competitive advantage to repopulation and engraftment.

Taken together, the accelerated development of leukemia in the absence of *Gadd45a* cannot be explained by differences in HSCs, homing and engraftment in non-oncogene expressing cells.

#### *Gadd45a*−/−/BCR-ABL cells exhibit hyperactivation of the PI3k/AKT, Stat5 and p38 pathway as well as increased expression of the dominant negative transforming isoform p30 C/EBPa, compared to WT/BCR-ABL cells

**Figure 6:**
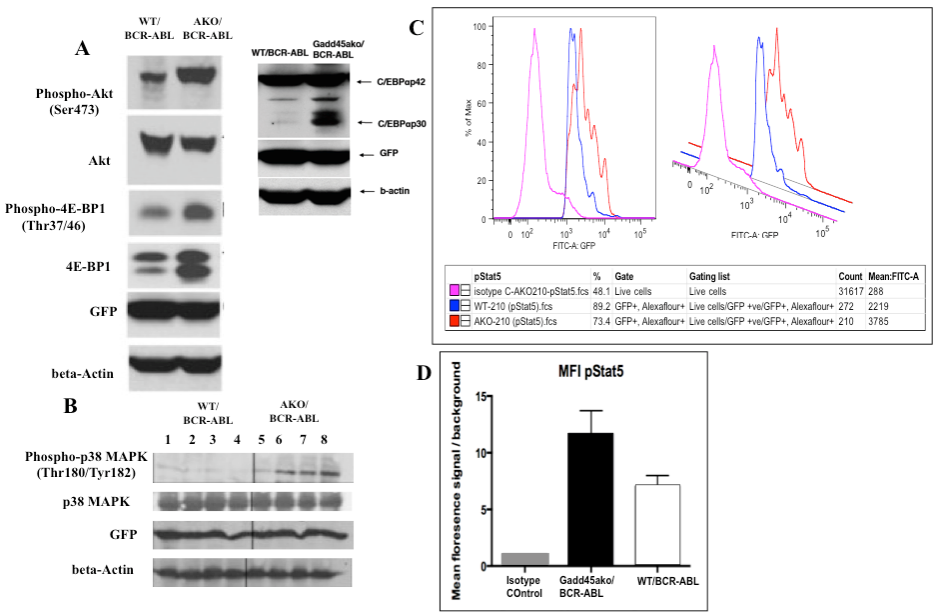
*Gadd45a*−/−/BCR-ABL cells exhibit hyperactivation of the PI3k/AKT, Stat5 and p38 pathway as well as increased expression of the dominant negative transforming isoform p30 C/EBPalpha **A**. *Gadd45a*−/− and WT BM cells transduced with BCR-ABL were cultured in myeloid expansion media for 11 days. GFP+ cells were obtained by sorting, proteins extracted as indicated in materials and methods, and immunoblotted with indicated antibodies **B**. Analysis of activation status of p38 MAPK in WT/BCR-ABL and *Gadd45a*−/−/BCR-ABL spleens. Spleens of recipient mice at 19 days post transplantation were prepared for western blot analysis as described in materials and methods. **C**.&**D**. Intracellular phospho-flow analysis of activation status of Stat5 using p-Stat5 conjugated Alexa flour 647 antibody *in vitro*. Isotype control was used to establish baseline reading. Mean fluorescence intensity of Isotype control, *Gadd45a*−/−/BCR-ABL and WT/BCR-ABL BM cells from **A**. were used. Background signals were established in the same populations by staining of matched isotype control.

To explore possible mechanisms by which *Gadd45a* suppresses CML, the activation status of various signaling pathways was assessed. Loss of *Gadd45a* in the presence of BCR-ABL led to hyperphosphorylation of AKT, with no change in total AKT. Additionally, it was observed that *Gadd45a*−/−/BCR-ABL cells exhibited hyperphosphorylation of 4EBP1 and increased levels of total 4EBP compared to WT/BCR-ABL (Figure 6A) (an important regulator of the translation machinery).

Importantly, it was observed that loss of *Gadd45a* in the presence of BCR-ABL resulted in increased expression of the dominant negative short isoform of C/EBPa p30 (Figure 6A); p30 C/EBPa has been reported to be associated with blocked granulocytic differentiation and aberrant progenitor proliferation of BM cells [29]. Additionally, mutated C/EBPa has been found in about 30% of Acute Myelogenous Leukemia (AML) cases and correlates with poor prognosis [30].

Gadd45a has been shown to interact with and modulate the function of p38 [1, 31]. To further understand CML disease pathogenesis in the absence of Gadd45a, p38 MAPK activation status was determined using protein lysates from spleens of CML mice. It was observed that p38 phosphorylation (p-p38) was consistently up-regulated in *Gadd45a*−/−/BCR-ABL cells, whereas total p38 levels were unchanged (Figure 6B) compared to WT counterparts

Stat5 plays a critical role in transformation of BCR-ABL-driven leukemia, since Stat5-abrogation either by gene deletion or expression of a dominant-negative mutant led to effective elimination of leukemia cells *in vivo* and *in vivo* [32] and [33]. In addition, Stat5 is consistently activated in BCR-ABL positive cell lines and in primary CML patients contributing to induction of cytokine independence [34]. Phospho-Stat5 levels were determined using intracellular phospho-flow analysis in BCR/ABL expressing BM. Figure 6C demonstrates 3 data peaks corresponding to isotype control (pink), WT/BCR-ABL (blue) and *Gadd45a*−/−/BCR-ABL cells (orange), indicative of the levels of phosphorylated Stat5, and thus of Stat5 activity. Quantification of p-Stat5 signals *via* calculation of mean fluorescence intensity (MFI) indicates that *Gadd45a*−/−/BCR-ABL BM cells exhibit increased phosphorylation/activation of Stat5, compared to WT/BCR-ABL BM cells (Figure 6D).

Although the signaling partners Ras and Jnk have been implicated as important downstream effectors of BCR-ABL driven CML [35] [36], no difference in the activation status of either Ras or Jnk pathways was observed (data not shown).

Taken together, these data show that hyperactivation of the AKT pathway as well as increased expression of transforming isoform p30 Cebpa, activation of p38 and upregulation of Stat5 pathway are associated with loss of Gadd45a in BCR/ABL-expressing hematopoietic cells.

### Gadd45a expression is up-regulated in a cohort of chronic phase CML patient samples but down-regulated in accelerated and blast crisis phase samples

Although public databases provide evidence for altered expression of Gadd45a in human CML, no information is provided about disease staging (data not shown). Therefore, Gadd45a expression in CML patients at different phases of the disease was determined to obtain a more complete assessment of a role for this potential tumor suppressor and possible diagnostic marker in a clinical setting.

**Figure 7:**
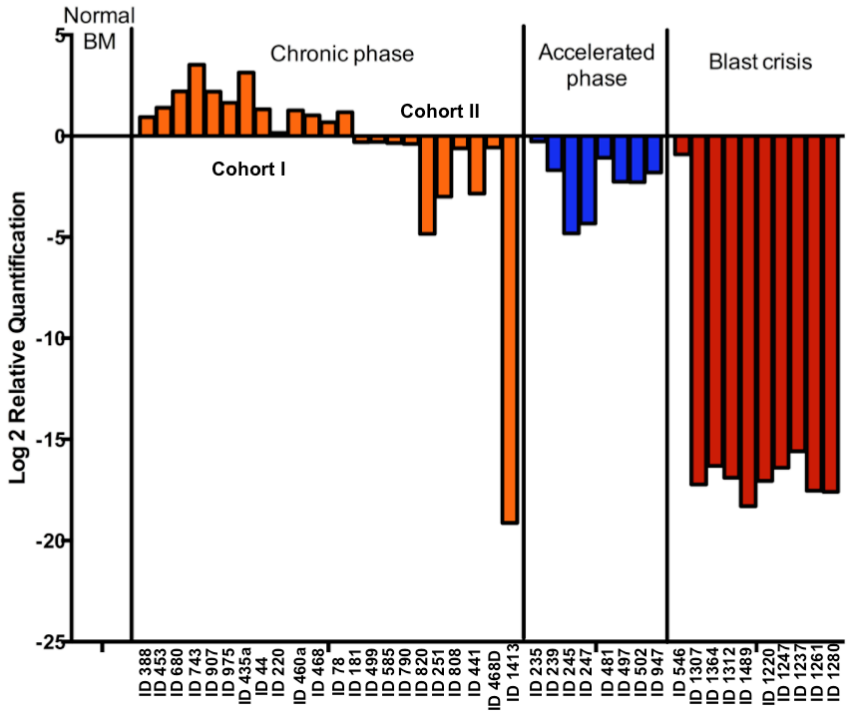
Gadd45a expression is up-regulated in a cohort of chronic phase CML patient samples but down-regulated in accelerated and blast crisis phase samples Gadd45a mRNA levels was measured in a panel of untreated human CML samples using Quantitative real time PCR analysis with taqman probe and expression was determined relative to 18s rRNA in AB Step one plus real time PCR machine. Plot shows normalized expression of *Gadd45a* in each CML cluster and in normal controls (CD34+ve BM, Peripheral blood and normal BM mononuclear cells) according to ΔΔCt method.

Real time PCR results from 23 chronic phase (CP), 8 accelerated phase (AP) and 10 blast crisis phase (BC) patient samples were compiled according to disease phase at the time sample was obtained. Interestingly, it was observed that Gadd45a expression is up regulated in a cohort of CP samples (cohort I) and is down regulated in all the AP and BC patient samples and in a cohort of CP samples (cohort II) (Figure 7). Notably, retrospective historical analysis of these patients revealed that all blast crisis patients that exhibited low levels of Gadd45a expression exhibited poor response to all forms of therapy resulting in the demise of most of these patients (data not shown). Further follow-up, using long-term longitudinal studies of CP (cohort II) and AP patients that exhibited low levels of Gadd45a expression, will establish if Gadd45a expression is an early indicator of disease progression.

Taken together, these data demonstrate that Gadd45a expression in human CML correlated with disease severity, where lower levels of Gadd45a in human CML suggest poor prognosis/survival. Collectively, this observation raises the possibility of using Gadd45a as a prognostic biomarker for disease staging and also raises the question of whether elevated levels of Gadd45a observed in initial phases of CML serve to impede disease progression.

## DISCUSSION

The current study highlights a unique role for *Gadd45a* as a tumor suppressor in BCR-ABL driven leukemogenesis, characterized by its’ loss causing an increase in the number and self-renewal capability of CML cells (Figure 4D). Additionally, it is shown that *Gadd45a* tumor suppressive function is associated with modulation of AKT, p38 and Stat5 signaling pathways, leading to deregulated cellular proliferation and apoptosis. Furthermore, our finding that Gadd45a expression correlates with human CML progression emphasizes the notion that *Gadd45a* can be used as a biomarker in patient samples and provides the framework for further longitudinal studies in order to correlate *Gadd45a* expression to CML prognosis.

It was shown that WT/BCR-ABL BM cells expressed significantly increased *Gadd45a* compared to WT-MIG cells *in vitro* (Figure 2A). Furthermore, in the presence of Imatinib, a BCR-ABL tyrosine kinase inhibitor, *Gadd45a* expression was reduced to baseline levels. This is in contrast to other known tumor-suppressor genes such as Pten and p53 [37, 38] that are downregulated by BCR-ABL. This may be due to the inherent function of *Gadd45a* as a stress response protein, wherein the presence of an oncogene results in its induction during the early stages of cancer development [3]. Whether *Gadd45a* expression is downregulated at a later time still needs to be verified with different time point experiments.

While this work was in progress, it was shown that Gadd45a plays a tumor suppressive role in other leukemias, such as FLT-3 and MLL-AF9 derived AML [17] [18]. Our data provides an important extension of this notion, showing for the first time that Gadd45a is a tumor suppressor in BCR-ABL driven leukemogenesis. These studies all support the tumor suppressive function of *Gadd45a* in broad-spectrum leukemia and point to targeting *Gadd45a* as an attractive therapeutic strategy that has the potential to have broad implications on a variety of hematopoietic diseases.

It is shown that *Gadd45a* deficiency in the presence of oncogenic BCR-ABL increases the number of leukemic stem cells (Figure 4C). Interestingly, Wingert et al., have shown that lentiviral transduction of LT-HSCs with GADD45A *ex vivo* leads to increased and accelerated differentiation into granulocyte-macrophage progenitor (GMP)-like cells [39]. Therefore, it would be interesting to further characterize the effect of Gadd45a on HSCs *versus* LSCs. Furthermore, our data suggest that enhanced *Gadd45a* expression may provide a means for eliminating BCR-ABL expressing LSCs, and thus targeting *Gadd45a* in combination with the BCR-ABL kinase inhibitor Imatinib could be an improved clinical approach to eliminate CML cells.

It is shown that hyperactivation of key signaling pathways, including the PI3k/AKT, Stat5 and p38 pathways, and expression of short transforming isoform of Cebpa in Gadd45a deficient BM cells may partly account for accelerated CML progression (Figure 6). It will therefore be interesting to study their functional hierarchy and relative contribution by performing pharmacological inhibitor studies.

While this work was in progress, evidence was obtained implicating Gadd45a in normal hematopoiesis and it was shown that at day 12 following 5-FU treatment, *Gadd45a*−/− mice had higher number of LSK cells compared to WT mice, while no difference in LSK cell numbers was observed at 5 days post 5- FU treatment [40]. Our data agrees with these findings providing evidence that the initial pool of BM cells obtained before transplantation experiments are similar (Figure 5C) and do not contribute to accelerated CML development.

Analysis of human CML samples revealed two distinct groups of chronic phase patients based on Gadd45a expression, where cohort I exhibited increased Gadd45a expression and cohort II exhibited reduced Gadd45a expression (Figure 7). It will be interesting to see if patients exhibiting reduced Gadd45a expression (cohort II) undergo faster and more aggressive disease development compared to cohort I patients. Given that mutations in the kinase domain (KD) are the most prevalent mechanism of BCR-ABL resistance in Imatinib treated cells leading to disease progression [41], it will also be interesting to determine whether lower expression of Gadd45a in chronic phase patients is a predisposing factor for acquisition of KD mutations post Imatinib treatment. If this is indeed the case, targeting elevation of Gadd45a expression along with tyrosine kinase inhibitors could hinder the development of BCR-ABL mutations and drug resistance.

Finally, it would be of interest to explore if overexpression of *Gadd45a* delays leukemia development, and whether other Gadd45 proteins (GADD45B and GADD45G) either separately or in combination with Gadd45a modulate CML development. Current research is targeted at addressing these interesting issues.

## MATERIALS AND METHODS

### Mice and genotyping

*Gadd45a*−/− mice (in a C57BL/6 129Svj background) were graciously provided by Albert Fornace (Georgetown University) and kept in specific pathogen-free animal facility at Medical School of Temple University. Ptprca Pepcb/BoyJ (CD45.1) mice were obtained from Jackson's Laboratory. Mice were genotyped by RT-PCR. PCRs using three primers allowed for simultaneous detection of the WT and mutant *Gadd45a* allele. These primers consisted of a 5′ upstream primer (5′-CACCTCTGCTTACCTCTGCACAAC-3′), a common 3′ downstream primer (5′-CCAGAAGACCTAGACAGCACGGTT-3′), and a neo-specific primer (5′-AAGCGCATGCTCCAGACTGCCTT-3′). Reactions were run for 37 cycles at 94°C for 1 minute, 63°C for 14 seconds, and 72°C for 12 seconds. All animal studies were approved by Temple University institutional animal use and care committee.

### Cell culture and cell lines

Myeloid progenitors which were isolated from the bone marrow of 5-fluorouracil (5-FU, 150mg/kg) treated mice were cultured in StemPro-34 SFM complete Medium (GIBCO Gibco, Gaithersburg, MD) or IMDM (GIBCO Gibco, Gaithersburg, MD) supplemented with 10% heat-inactivated horse serum (Gibco), 1% penicillin- streptomycin (Gibco) and cytokine cocktail SCF (100ng/ml), IL-3 (20ng/ml), IL-6 (20ng/ml) and Flt-3 (100ng/ml). For *in vitro* molecular signaling detection, the concentration of the cytokines were as follows: SCF (12ng/ml), IL-3 (5ng/ml), IL-6 (5ng/ml). Human K562 myeloid leukemia cell line was grown in RPMI 1640 medium containing 10% FBS. All cells were maintained in a humidified atmosphere with 10% CO_2_ at 37°C.

### BCR-ABL-expressing retroviral vector and bone marrow transplantation

The retroviral vector MSCV-BCR/ABL-IRES-GFP and retroviral transduction /transplantation of mouse bone marrow cells for induction of CML by BCR-ABL has been described previously [22]. MSCV-IRES-GFP and MIGR1-BCR-ABL-GFP was a gift from Warren Pear [25]. Phoenix E packaging cells (Orbigen, Inc) or Gryphon cells (allele biotech) were transfected with either control MIGR1 or MIGR1-BCR-ABL vectors using calcium phosphate precipitation method. 48 hours after transfection, high-titer, helper-free, replication-defective ecotropic virus stock was harvested and centrifuged at 3200rpm for 10 minutes at 32°C. Myeloid progenitor enriched bone marrow (BM), cells obtained from WT and *Gadd45a*−/− mice treated with 5-Fluorouracil (5-FU) were infected with *empty vector* MIGR1 or MIGR1-BCR-ABL virus. Specifically, 3ml of retroviral supernatant supplemented with 10µg/ml polybrene (Sigma-Aldrich) was added to 1×10^6^ cells and spinoculated at 2200rpm for 45 minutes at 32°C. The infection efficiency was determined on the basis of percent GFP+ve cells by flow cytometry. The syngeneic wild type recipient mice (6-12 weeks old) were lethally irradiated with 900 rads (^137^Cs source). 5000 GFP positive cells along with 495,000 GFP negative accessory cells were introduced into lethally irradiated syngeneic WT recipient mice by retro-orbital injection.

### FACS analysis of normal hematopoietic stem cells (HSC), leukemic stem cells (LSC) and cell sorting using flow cytometry

Hematopoietic cells were collected from the bone marrow and peripheral blood of the normal and diseased mice. Erythrocytes were lysed in NH_4_Cl red blood cell lysis buffer (pH 7.4). The cells were washed with PBS and stained with Gr-1-APC for neutrophils, B220-PE for lymphocytes, F480-APC for macrophages and Sca-1/c-Kit/Lin for HSC purchased from Ebioscience Inc or cell signaling. After staining, the cells were washed once with PBS and subjected to FACS analysis. Cells were analyzed with FACS calibur or LSRII (Becton Dickinson). Influx cell sorter (Becton Dickinson) was used for sorting of GFP positive cells from cultured myeloid progenitors.

### Cell proliferation assay by BrDU analysis and analysis of apoptosis by annexinV

The BrdU incorporate assay was performed using BrdU-APC flow kit according to the manufacturer instruction (BD Pharmingen). CML mice were injected with 10mg/ml BrDU. 24 hrs post injection, bone marrow cells were extracted from CML mice and analyzed by flow cytometry. Apoptosis of bone marrow cells was measured by using the Annexin V-APC apoptosis detection kit II along with 7AAD staining for cell viability, according to the manufacturers instruction (BD Pharmingen. Differentially labeled cells were resolved using flow cytometry (BD FACS Calibur) and analyzed with Flowjo 5.2 software (Tree Star). Cells stained positive for Annexin V-APC and negative for 7AAD represented cells in early stage of apoptosis. 10,000 events from each sample were acquired to ensure adequate data.

### Colony-forming assay

The methylcellulose colony forming assays were performed with 2×10^4^ GFP+ve BM cells expressing BCR-ABL. Cells were suspended in Methocult medium (StemCell Technologies) and cultured on 3-cm diameter dishes according to manufacturer's instructions. After 7 days colonies were counted and harvested, and 2×10^4^ cells were replated for 2^nd^ and 3^rd^ rounds.

### Analysis of cell morphology

Cytospins were stained with May-Grunwald-Giemsa, and observed under bright field microscopy (Olympus AH-3; Tokyo, Japan) using a 20x/0.8NA objective. Images at low magnification (200x) were acquired through an Insight camera (Diagnostic Instruments Inc, Sterling Height, MI) using the imaging software SPOT (Diagnostic Instruments Inc) in order to analyze for morphologic differentiation [26, 27]. Results of all experiments represent the mean of at least 3 independent determinations.

### Drug treatment

Imatinib (LC laboratory, Woburn- MA, Cat no 15508) was dissolved in water to a stock concentration of 1 mg/ml. Further dilutions were made to working concentrations using media or water.

### Protein extraction and immunoblot analysis and intracellular phospho flow analysis

Total protein lysates were prepared by resuspending bone marrow cell pellets at a concentration of 10^7^/ml in CST-cell lysis buffer (Cell Signaling technologies, MA). Protein concentration was determined using Bio-Rad protein assay (Bio-Rad) followed by spectrophotometer readout at a wavelength of 595-nm. Sixty micrograms of each sample was fractionated on SDS-PAGE gels. Resolved proteins were transferred to PVDF membrane (Millipore) using a Trans-Blot apparatus (Bio-Rad) at 60V for 1 hour. Equal loading of protein was verified by staining for β-actin protein. Blots were probed with the appropriate primary antibody overnight at 4°C in TBS and 0.1% Tween-20. After incubation in primary antibody, the blots were probed with corresponding secondary antibody conjugated to horseradish peroxidase (Cell Signaling Technology, Inc). Detection of protein bands was developed using chemiluminescent Western detection system (LumiGLO, Cell Signaling). Primary antibodies against c-abl, AKT, Cebp, ERK, GFP, p38, Stat5, Nras and β-actin were from Cell Signaling Technology, Inc. (Danvers, MA). Intracellular phospho flow analysis was performed as previously described [28]. Cells were harvested with HBSS and washed 2 times by adding 2 mL of PBS (or HBSS), centrifuging at 300 x g for 5 minutes, and then decanting buffer from pelleted cells. 1 × 10^6^ cells/100 μL were aliquoted into FACS tubes and 0.5 mL of cold Flow Cytometry Fixation Buffer and were incubated at room temperature for 10 minutes in order to maintain a single cell suspension. Phospho Stat5 rabbit antibody (Cell Signaling, Cat number 9359) was added at 1:100 dilution and Alexa fluor conjugated anti rabbit IgG (H+L),F(ab’)_2_ fragment was added as a secondary antibody. FACS-calibur and Flowjo was used to read and interpret data respectively.

### Real time PCR analysis

Total RNA was isolated using Qiagen RNA extraction kit and reverse-transcribed by the ThermoScript RT-PCR system (Invitrogen) with an oligo-dT primer. Real-time quantitative PCR (qPCR) was performed with an ABI Prism 7300 Thermal Cycler (Applied Biosystems) using taqman probe (Roche) and expression was determined relative to 18s rRNA (endogenous control) in AB Step one plus real time PCR machine. Probes used were the following, all purchased from Life Technologies: Hs99999901_S1 (human 18S), Hs00169255_m1 (human Gadd45a), Mm00432802_m1 (mouse Gadd45a) and Mm04277571_S1 (mouse 18S).

### Histology

Spleen and liver were dissected from moribund mice and fixed in a 4% formaldehyde solution immediately. Slides from these fixed organs (prepared by Histotechnology Core, The Wistar Institute) were stained with Hematoxylin & Eosin (H&E) and examined using an Olympus inverted microscope with digital imaging.

### Human samples

Peripheral blood (PB) or Bone marrow (BM) samples from a panel of CML patients were obtained from Dr. Ravi Bhatia (City of Hope National Medical Center). Normal frozen samples (0.3×10^6^ - 5×10^6^) consisting of Peripheral blood, Bone marrow mononuclear cells and CD34+ve cells were obtained from the University of Pennsylvania School of Medicine and Stem Cell Technologies. Sample acquisition was approved by the Institutional Review Board at the COHNMC and met all requirements of the Declaration of Helsinki.

### Statistics

Statistical analyses were performed by using Student t Test (*: *p* < 0.05, **: *p* < 0.01) (GraphPad Prism v5.01 software for Windows, GraphPad Software, San Diego, CA
